# Thomas Luckmann on the Relation Between Phenomenology and Sociology: A Constructive Critical Assessment

**DOI:** 10.1007/s10746-021-09577-4

**Published:** 2021-04-22

**Authors:** Alexis Gros

**Affiliations:** 1 Friedrich-Schiller-Universität Jena, Jena, Germany; 2 University of Buenos Aires, Buenos Aires, Argentina; 3 CONICET (National Scientific and Technical Research Council), Buenos Aires, Argentina

**Keywords:** Thomas Luckmann, Phenomenology, Sociology, Phenomenological sociology, Proto-sociology

## Abstract

In the present paper, I intend to systematically revisit Thomas Luckmann’s account of the relation between phenomenology and sociology and to assess its strengths and weaknesses in terms of constructive criticism*.* In order to achieve this aim, I will proceed in three steps. First (1), I will reconstruct the Luckmannian approach by means of an exhaustive analysis of his programmatic texts. Second (2), I will identify its strengths and merits. And finally (3), I will discuss its shortcomings and try to correct them in two ways, namely, by bringing Luckmann into a dialogue with other sociological-theoretical and phenomenological accounts, and by counteracting some of his problematic claims with alternative insights contained in his own work.

## Introduction

It’s been many years since I have formulated the program of proto-sociology […] for the first time. As far as I know, this program has not been much discussed, let alone criticized in a constructive manner.

Thomas Luckmann (1993: 321).

Alfred Schutz’s work is undoubtedly the most influential reference when it comes to thinking about the relation between phenomenology and sociology. In his 1932 book *Der sinnhafte Aufbau der sozialen Welt* (Schütz 1991 [1932]) and in numerous papers published during the 1940s and the 1950s (for instance, Schutz 1962b [1945]), he provides what is probably the most thorough treatment available of the issue, and this on the basis of an exhaustive knowledge of both research fields. For this reason, Schutz is considered worldwide as the founding father and key figure of phenomenologically oriented sociology (see, for example, Endreß and Srubar 1997: 651; Belvedere 2011; Zahavi 2018: 106).

Accordingly, it is not surprising that, at least since the 1960s, the whole debate concerning the relationship between these two disciplines centers around the interpretation and further development of the Schutzian legacy (Eberle 2012b: 135). This debate is usually depicted as marked by an antagonism between two opposed positions, each of which is associated with a geographical location, namely, the US-American approach, prominently represented by George Psathas,^1^ and the German one, organized around the figure of Thomas Luckmann (Eberle 2012b: 135f., 148; Dreher 2012: 11f.).^2^ Schematically speaking, while the former argues for a synthesis between empirical sociology and phenomenology in terms of a “phenomenological sociology,” the latter understands this relation rather as a cooperation between two “different” research fields that should not be conflated with each other (Eberle 2012b: 135).

Although Luckmann is a well-known sociological figure worldwide,^3^ his *programmatic* account of the relation between phenomenology and sociology, expounded in various articles published during the 1970s, 1980s, and 1990s (see, for instance, Luckmann 1979; 1983a [1973]; 1991), seems to be barely known and discussed outside the German-speaking world.^4^ Indeed, his criticism of the popular label “phenomenological sociology” and his alternative conception of phenomenology as a “proto-sociological” approach are mostly neglected both in the English-speaking and the Spanish-speaking debates,^5^ being only thematized either in German-speaking literature (see, for example, Endreß and Hahn 2018; Schnettler 2006) or in English texts written by German-speaking scholars (see, for instance, Knoblauch 2011; Dreher 2009).

Whether one agrees with Luckmann’s account of the relationship between the two disciplines or not, it would be folly to deny its value: it provides a serious and well-thought-out take on this complex issue that cannot be simply ignored. In this sense, its neglect both in the English- and Spanish-speaking literature seems to be a sufficient reason for my attempt here to systematically revisit it. Arguably, however, the need for a thorough reconstruction and assessment of the Luckmannian position also finds justification in three other reasons.

First of all, Luckmann’s polemical and categorical style in his most programmatic texts (1983a [1973]; 1979; 1991; 2007c [1999]), i.e., his tendency to use strong formulations, clear-cut definitions, and schematic antinomies, constitutes sometimes an obstacle for gaining an adequate understanding of the many nuances involved in his account. I think this problem can be tackled by means of a *hermeneutic-reconstructive effort*, that is, by elucidating his programmatic formulations in light of a holistic view of his work informed by the existing secondary literature.

Second, the fact that most literature on Luckmann was written by German-speaking sociologists of knowledge who either directly or indirectly worked with him (see, for instance, Schnettler 2006; Knoblauch 2011; Endreß and Hahn 2018) makes it difficult for “outsiders” to get a comprehensive insight into his approach. I say this from my own experience as a South American sociologist and phenomenologist working in Germany. As Schutz (1964 [1944]) rightly notes in his classic essay “The Stranger,” outsiders normally face difficulties when attempting to grasp the schemes of interpretation prevalent in a foreign group. For group insiders, and this also applies for the members of scientific groups, certain things require neither explication nor articulation: they are so familiar that have become taken-for-granted. I think my attempt here to reconstruct and discuss Luckmann’s programmatic account from a fresh, outsider perspective could help clarify and articulate some aspects thereof that are not sufficiently explained in the available secondary literature.

And third, and most importantly, the relative absence of exhaustive *constructive criticisms* of the Luckmannian account in the German-speaking literature justifies the need for an assessment of its merits and demerits.^6^ In this sense, I believe the complaint expressed by Luckmann (1993: 321. My emphasis) himself in a 1993 article remains highly topical: “It’s been many years since I have formulated the program of proto-sociology […] for the first time. As far as I know, this program has not been much discussed, let alone *criticized in a constructive manner*”.

Against this background, in the present paper I intend to provide a systematic reconstruction of Luckmann’s programmatic account and an assessment of its strengths and weaknesses. More precisely, my objective here is to reconstruct, present, and analyze its main features, thereby identifying both its merits and shortcomings, and subjecting the latter to *constructive criticism,* i.e., to a critical examination that does not aim at dismounting the Luckmannian approach but, rather, at revising and further developing it. In doing so, I start from the following thesis: although some of Luckmann’s tenets must be nuanced, his position constitutes a solid and promising approach to applying phenomenological insights in sociology.

In order to achieve the stated aim, I will proceed in three steps. First (1), I will reconstruct Luckmann’s account of the relation between phenomenology and sociology by means of an exhaustive analysis of his programmatic writings. Second (2), I will assess its strengths and merits. And finally (3), I will identify its shortcomings and try to correct them in two ways, namely, by bringing Luckmann into a dialogue with other sociological-theoretical and phenomenological accounts, and by counteracting some of his problematic claims with alternative insights contained in his own work.

## Towards a “Parallel Action”: A Systematic Reconstruction of Luckmann’s Programmatic Account

### The Strict Separation Between Phenomenology and Sociology

Luckmann sees phenomenology and sociology as two disciplines that can, and should, cooperate with each other but without ever losing their respective identities and boundaries. More precisely, on the one hand, he emphasizes the relevance of phenomenological philosophy for the epistemological foundation of sociology (Luckmann 1979: 199; 2007b [2001]: 139). But on the other hand, he argues that they constitute two divergent analytic approaches that must be strictly differentiated. As he writes, “[t]he differences in perspective, cognitive style and method between these two modes of knowledge should not be confounded” (Luckmann 1978: 11; see 1979: 197; 2008: 33).

In this sense, he defends a thesis that may sound surprising for those not familiarized with his work, namely, that the idea of a “phenomenological sociology,” endorsed by authors such as George Psathas (1973, 1989), constitutes a “conceptual contradiction” [*begrifflicher Widerspruch*] (Luckmann 1979: 205). As Luckmann polemically puts it in a passage from the article “Phänomenologie und Soziologie”: “there is no such thing as a phenomenological sociology […]. [S]uch thing will never exist” (1979: 196).

Luckmann (1979: 196; 2002a [1990]: 139), thus, acknowledges the possible contributions of phenomenology to the philosophical underpinnings of sociology but, at the same time, criticizes the attempt of fusing both disciplines into a hybrid construct such as a “phenomenological sociology”. Phenomenology, he claims, constitutes a philosophical approach and, as such, should not be conflated with an empirical method of social research (Luckmann 1979: 196; 2002a [1990]: 139). It is “not a field of *research*” but, rather, a “field of *thought* […], analysis or philosophical description” (Luckmann in Dreher and Göttlich, 2016: 28f.).

Against this background, Luckmann (1979: 197; 2007c [1999]: 131) depicts the relation between both disciplines metaphorically as a “parallel action” [*Parallelaktion*] (see Dreher 2009: 404).^7^ Just like two parallel lines, phenomenology and sociology run in the same direction but never really meet: “The great significance of phenomenology for the social sciences is due to the fact that it constitutes a theoretical enterprise that *runs parallel* to the empirical sciences, rather than being identical with them” (Luckmann 1979: 197. My emphasis).

### Differences Between Science and Phenomenology

According to Luckmann (1979: 196ff.; 1991: 155f.), the main difference between phenomenology and sociology is that the former constitutes a “philosophy” [*Philosophie*], while the latter counts as a “science” [*Wissenschaft*]. Following the “habitual” view stemming from the natural sciences, he argues that all sciences aim at theoretically “reconstructing” reality, or, more precisely, a segment of it. That is, they attempt to describe *and* explain the objective world through methods that are both logical-rational and empirical (Luckmann 1983a [1973]: 17). “[E]very modern empirical science [*Erfahrungswissenschaft*],” he writes, “is an attempt to systematically reconstruct reality” (Luckmann 2007a [2003]: 162).

In line with Karl Popper (2002 [1959]: 15), Luckmann (1991: 155; see 1979: 197; 1989: 27) maintains that sciences are “cosmological” disciplines: they are guided by the “common interest” of “*understanding*” (Popper in Luckmann 1991: 155) and explaining the workings of the world as a means of enabling and facilitating human praxis (Luckmann 1983a [1973]: 17). For this very reason, they cannot help being “naively” realistic. Their “fundamental assumption” is that there is an external reality “out there” which can be theoretically reconstructed (Luckmann 1989: 27).

More specifically, Luckmann (1979: 198; see 1991: 155) claims that the “main objective” of sciences *qua* cosmological disciplines consists in “explaining the general features” of the “objective world”. And they achieve this aim, he says, by means of a method that is both “empirical” and “rational” (Luckmann 1983a [1973]: 17). On the one hand, scientific knowledge is empirically gained on the basis of “‘inductive’ generalization” (Luckmann 1979: 198); and on the other, it is characterized by a high degree of explicitness, systematization, and formalization, all features that ensure not only its accuracy but also its transmissibility, public verifiability, and controllability (Luckmann 1979: 201; 1983a [1973]: 17).

In Luckmann’s view, phenomenology *is not* a science in the latter sense. Although it operates with a systematic, logical-rational, and, in a very specific sense, empirical method, it is not cosmological, realistic, and inductive but, rather, *egological*,^8^*reflective, and ontologically neutral* (Luckmann 1979: 196f.)*.* As he writes in a 1991 paper: “First, the phenomenological perspective is egological, not mundane-realistic. Second, its method is egological-reflective [*bewußtseins-‚reflexiv’*], not realistic-inductive [*wirklichkeits-‚induktiv’*]. And third, its aim is not immediately cosmological” (Luckmann 1991: 156).

More specifically, phenomenology is for Luckmann (1979: 196; 1991: 155ff., 163) a *philosophical* discipline aiming at *describing* the a priori,^9^ i.e., universal, necessary, and historically invariant, structures of *subjective* “experience” [*Erfahrung*], and this by means of methodically controlled *reflection*^10^ upon one’s own consciousness*.* In a “Cartesian” vein, says Luckmann (2007c [1999]: 128), phenomenological research starts from the immediate “evidence” of one’s own consciousness and proceeds methodically to “*describe the universal structures of the subjective orientation in the world*” (Luckmann 1979: 198) –e.g., the structural features of attention, perception, action, lived temporality and spatiality, intersubjectivity, etc. (see Luckmann 1983a [1973]: 32, 42).

To put it even more precisely, according to Luckmann (2007c [1999]: 128), phenomenology investigates the basal qualities of “*intentional*” *“*conscious activities” [*Bewusstseinstätigkeiten*], these understood as lived experiences meaningfully directed towards something “outside”^11^ consciousness, namely, to “intentional objects” or *noemata* of different kinds (Luckmann 2008: 34). On this Husserl-inspired account, “consciousness *itself* is ‘nothing’, except […] consciousness *of*” (Luckmann 1989: 20).

Phenomenology’s fundamental claim, says Luckmann (2008: 34), is that the “lifeworld” [*Lebenswelt*], i.e., the experiential environment in which we live and act as pre-scientific actors, “constitutes itself” *qua* meaningful-experiential reality in our intentional experiences. This means that the fundamental structures of our pre-scientific world are allowed to appear the way they do thanks to the meaning-giving, intentional activities of our consciousness. “Phenomenological research, from Husserl to Gurwitsch and Schutz, has shown how the general structures of the lifeworld are constituted in certain conscious activities” (2008: 34).

As said, for Luckmann (2007b [2001]: 141; 1979: 198), phenomenological “constitutive analysis” [*Konstitutionsanalyse*] aims at describing and articulating the universal, necessary, and invariant features of the intentional constitution of our world-experience. In order to work out these a priori structures, says Luckmann (1979: 198), it is necessary to apply two methodological “reductions” [*Reduktionen*] originally developed by Husserl (Hua III: §§ 31–32; 1972: § 87): the “*epoché*”^12^ and the “eidetic variation”. With the help of these methods of “controlled abstraction” (Hitzler and Eisewicht 2020: 14), phenomenologists are able to proceed from the *concrete*, i.e., biographically, socio-culturally, and historically shaped, experience to its “formal structures” or “fundamental strata [*Schichten*]” (Luckmann 2008: 38; see 1983b [1970]: 41).

The constitutive analysis developed by Husserl, operating with the *epoché* […] and the eidetic variation […], shows that no matter from which concrete experience one starts, it is possible to differentiate its biographically and historically modifiable, concrete constituent parts from those “formal” structures without which that concrete experience would be unthinkable. (Luckmann 2008: 35)

Following Husserl (see Hua III: §§ 31–32), Luckmann (1979: 197, 205; 2007b [2001]: 140f.) characterizes the *epoché* as an attempt to suspend the assumptions and prejudices that occlude our “immediate” intentional experience and its constitutive achievements. On the one hand, the *epoché* brackets the “claim to reality” [*Wirklichkeitsanspruch*] of our lifeworldly experience, that is, the naïf belief, prevalent both in pre-scientific and scientific thinking, that reality simply exists “out there,” independently of our intentional activities (see Luckmann 1979: 197). And on the other hand, it neutralizes the validity of *all* theoretical presuppositions concerning the nature and origins of mundane phenomena (Luckmann 2007b [2001]: 140f.). After performing this “stepwise exclusion [*Ausklammerung*] of strata of meaning” (Luckmann 2007c [1999]: 129), the phenomenologist is able to *observe and describe her experience just as it immediately presents itself* (Luckmann 2007b [2001]: 140f.; 1979: 197; 1983a [1973]: 29).

Although Luckmann (see, for instance, 2007c [1999]: 129) does not offer an exhaustive definition of the eidetic reduction, he seems to understand it in a similar manner than Husserl (1972: § 87), namely, as an attempt to carve out the *essential* layers of lifeworldly experience by means of a “thought experiment” (see Wiesing, 2009: 101ff.). The method of eidetic reduction, writes Luckmann (2007c [1999]: 129), implies an “imaginary variation of that what is consistently possible”: the phenomenologist takes a concrete phenomenon as a starting point and modifies it freely in imagination until she finds those structural features without which that kind of experience “would be unthinkable” (Luckmann 2008: 35).

### Luckmann’s Weberian Conception of Sociology

For Luckmann (1979: 198; 2008: 33; 2007b [2001]: 141; 1989: 17), sociology is a cosmological empirical science. Just like biology, chemistry or physics, it aims at systematically reconstructing a part of objective reality, namely, the social world, by means of empirical-rational methods. Unlike nature, however, the social world is not a mute, physical reality but, rather, a “*human* reality,” that is, a meaningful world historically “*constructed*” [*konstruiert*] in everyday (inter)actions of human subjects (Luckmann 2008: 33. My emphasis; see 1989: 17). “The human world,” says Luckmann (1978: 10f.), “is a man-experienced world and […] a man-made world”. From this peculiarity of social reality follows that the methodological strategies of the natural sciences are untenable in sociological research (Luckmann 2007b [2001]: 141).

More precisely, when specifying the nature of sociology *qua* science, i.e., its subject matter and methodology, Luckmann (2007b [2001]: 139) draws heavily on the tradition of “interpretive sociology” [*verstehende Soziologie*] founded by Max Weber and continued by Schutz. According to the Weberian approach as read by Luckmann (2002a [1990]: 53), sociology is an “empirical science” [*Erfahrungswissenschaft*] (see Weber 1984: 148) that aims at “describing” *concrete* socio-historical worlds and at casually “explaining” their genesis, and this by means of “understanding” [*Verstehen*] the human actions through which they are or were constructed (Luckmann 1979: 199; 2008: 33; 2007c [1999]: 131). “For Weber,” writes Luckmann (1979: 199), “sociology is a science that *explains* human behavior through the *interpretation* of human action”.

On Luckmann’s Weber-inspired account, the human actions which construct social reality are neither mechanically caused by biological instincts nor blindly determined by objective social structures. Rather, they are motivated and oriented by subjective interpretations or “pre-scientific operations of understanding [*Verstehensleistungen*]” performed by the actors themselves (Luckmann 2007a [2003]: 152). To be sure, subjective interpretations do not emerge *ex nihilo*. On the one hand, and this is crucial, they necessarily rest upon the “universal” structures of subjective, intentional experience worked out by phenomenology (Luckmann 2007c [1999]: 136). And, on the other hand, they are always already made possible, mediated, and restricted both by culturally imposed “stocks of meaning” [*Bedeutungsbestände*] and social-institutional settings (2007c [1999]: 136).

Now, although these socio-cultural structures tend to appear to actors as objective and unmodifiable things (see Berger and Luckmann, 1966: 1, 59), they are nothing but objectivations of everyday social action, that is to say, “partly intended, partly unintended results” thereof (Luckmann 2007c [1999]: 136f.). Accordingly, Luckmann defines the social “construction” of reality as a “goal-oriented human and social activity under contingent limiting conditions” (2007c [1999]: 137).

To use an expression by Ilja Srubar (2007: 66), in this perspective social reality appears as a “*meaningfully structured plexus of actions*” [*sinnstrukturierten Handlungszusammenhang*]. As Luckmann (2007a [2003]: 163; see 1979: 204; 1983a [1973]: 28) in line with Schutz (see 1962a [1953]: 5) emphasizes, the pre-theoretical interpretations of everyday actors are not a “contingent element” of social reality but, rather, a “constitutive component” thereof. While natural scientists study and interpret objects that do not possess any *immanent* pre-scientific meaning –atoms, molecules, cells, etc.–, social scientists deal with “pre-constituted” [*vorkonstituiert*] “data,” i.e., with a reality always already pre-interpreted by lay actors.

In other words, the “data of the social sciences” is meaningful for human beings “before and after” sociological research (Luckmann 2007a [2003]: 163; see 1989: 19, 28). In order to do justice to this key ontological feature of its subject matter, sociology is obliged to operate with a *hermeneutic-reconstructive* method: “Thus one may say that in sociology historical hermeneutic reconstruction comes before ‘measurement’ or –in Weberian terms—interpretation comes before explanation” (Luckmann 1989: 28). More precisely, by means of complex “operations of understanding,” claims Luckmann (2007a [2003]: 163) in line with Schutz (see 1962a [1953]: 44), sociologists attempt to elaborate adequate “second-order” reconstructions of the “first-order meaning constructs” of everyday actors (Luckmann 1989: 19).

Against this background, the Luckmannian claim that the task of sociology consists in the “*empirical* scientific *reconstruction* of historical human constructions of reality” becomes understandable (Luckmann 2008: 35. My emphasis). For Luckmann, sociological empirical research intends to “describe” *particular* socio-historical phenomena or worlds and to “explain” their specific historical genesis (see Dreher 2009: 405). In order to do so, it resorts to classical research methods, such as ethnographic fieldwork or interviews (see Luckmann in Dreher and Göttlich, 2016: 29ff.).

### Phenomenology and Sociology: Differences, Compatibility, and Relation of Foundation

Luckmann (1979: 197; 2008: 35; 2007b [2001] 139f.) characterizes phenomenology and sociology as two different “forms” or “planes of thinking” [*Denkweisen/ Denkebenen*] regarding “human realities,” that is, as two ways of investigating social phenomena. On the one hand (a), one can *sociologically reconstruct* the intersubjective construction of a particular socio-historical phenomenon by means of empirical qualitative methods. And on the other hand (b), with the help of *phenomenological constitutive analysis*, it is possible to carve out the a priori structures of subjective experience upon which all factual processes of social construction are based (see Luckmann 2008: 35; Dreher 2012: 11f.).

For instance, one can analyze a concrete socio-historical phenomenon, such as the “virtual” intersubjective relationships via *Skype* and *Zoom* that have become prevalent worldwide during the Coronavirus crisis in 2020, either (a) *sociologically* or (b) *phenomenologically*. With the help of qualitative methods of empirical research (a), such as interviews, one attempts to hermeneutically reconstruct, and thereby explain, the lived experiences of individuals using video calling apps for chatting with friends, working, studying, etc. In contrast, by means of phenomenological reflection (b), one seeks to “reduce” those biographically and historically concrete experiences to the basal strata of intentional consciousness on which they rest, i.e., to the invariant structures of empathy, consciousness of image, etc. (see, for example, Olser 2021). In the first case, one operates with the concept of “*construction*” [*Konstruktion*], while in the second case the notion of “constitution” [*Konstitution*] plays the leading role (Luckmann 2007c [1999]: 129; see Dreher 2012).

Importantly, for Luckmann (1979: 199ff.), these two forms of thinking are *different but compatible*. The *egological* and *meaning-theoretical* orientation of phenomenology has elective affinities, as it were, with the *methodological-individualistic* and *hermeneutic* nature of interpretive sociology (Dreher 2012: 10; Eberle 2012b: 138). It is precisely this compatibility that opens up the possibility of establishing a relation of cooperation between both disciplines in terms of a “parallel action” (Dreher 2012: 10f.).

More specifically, inspired by Schutz (see 1962b [1945]: 118), Luckmann (1979: 199; 1991: 159, 162) conceives the relationship between phenomenological and sociological research as a relation of “*foundation*” [*Fundierung/ Begründung*]. Phenomenology, he says, should provide the “philosophical,” or better, *proto-theoretical*, basis for sociology. In his own words: “Sociology as an empirical science starts where phenomenology, as the possibility of its philosophical foundation, ends” (Luckmann 2002a [1990]: 53).

### Phenomenology as a Proto-Sociological “Mathesis Universalis”

In line with his strict conception of scientific knowledge, Luckmann believes that each science must rest on a *proto-theory*, that is, on a “rational and systematic understanding” (Luckmann 1991: 159) of its subject matter crystallized into a “formal theoretical language” (Luckmann 1979: 202). By means of general concepts, this “proto-language” [*Protosprache*] must explicitly define the “domain” of the scientific discipline, i.e., its “field” of study and the “constituent elements” thereof (Luckmann 1983a [1973]: 22ff.).

More precisely, on Luckmann’s (1979: 204; 1983a [1973]: 21, 26) account, a scientific proto-theory operates as a “theory of measurement,” insofar as it allows for a “systematization” and “formalization” of the processes of “observation,” “identification,” and “classification” of empirical data. With the help of this proto-language, researchers know exactly *what* to attend to in the empirical field and *how* to “measure” or categorize it. This proves to be crucial for ensuring the accuracy, verifiability, and comparability of scientific results (Luckmann 1983a [1973]: 27f.).^13^

According to Luckmann (1983a [1973]: 35), the natural sciences find such proto-theory in the so-called “Copernican-Galilean-Newtonian view of the world”. The latter can be characterized as a “*mathesis universalis,*” insofar as it conceptualizes “nature” as a purely physical reality which is ultimately mathematically structured (Luckmann 1983a [1973]: 10, 16). “In the natural sciences,” one reads in a 1979 paper, “the formal theoretical language stems from mathematics” (Luckmann 1979: 202).

Yet, as “successful” this mathematized proto-language may be for the study of physical reality, all attempts of applying it to social research are, from the outset, condemned to failure (Luckmann 1991: 158; 1983a [1973]: 15). Indeed, as Luckmann (1983a [1973]: 15) rightly argues, basing the social sciences upon such naturalistic proto-theory entails disregarding, and even adulterating, the meaningful nature of social reality. Unfortunately, social scientists lack an alternative language adequate to their subject matter and, as a consequence, are immersed in a severe epistemological “crisis” (1983a [1973]: 19).

In Luckmann’s view, what social scientists in general and sociologists in particular lack is a “proto-sociology” [*Protosoziologie*],^14^ that is, a general “interpretative framework” [*Deutungsrahmen*] for conceptually apprehending, analyzing, and “measuring” social reality (Luckmann 1991: 165; 1979: 202f.). The social sciences, he argues, have been hitherto unable to solve the problem of the “systematization” and “formalization” of their domain of research (Luckmann 1983a [1973]: 15, 19; 1979: 201). Put differently, there is no established *“mathesis universalis* appropriate to the social world” (Luckmann 1983a [1973]: 19).

As a consequence, empirical social researchers tend to operate with an unacceptable “methodological naivety” (Luckmann 1983a [1973]: 27). Lacking an adequate proto-sociology, they unreflectively resort to “*common sense* taxonomies” when it comes to identifying, measuring or conceptualizing the observed material (Luckmann 1983a [1973]: 27). This, in turn, results in the production of imprecise and unreliable data which are either ethnocentrically biased or naturalistically truncated (Luckmann 1979: 203).^15^

As suggested above, Luckmann (1991: 159) argues that Weber took an important step towards developing a non-reductionist “proto-theory” for the social sciences. However, he did not go far enough: Weberian interpretive sociology fails at offering an exhaustive analysis of the fundamental structures of social reality, and this because it does not provide a thorough account of the processes of (inter)subjective “meaning-constitution” [*Sinnkonstitution*] through which it is constructed (Luckmann 2002b [1982]: 120; 1991: 159). It is Alfred Schutz (see 1991 [1932]: 9 ff.), with his attempt of *phenomenologically* correcting this deficit, who makes the decisive step forward in elaborating a truly “meaning-adequate” [*sinnadäquat*] proto-sociology (Luckmann 1979: 199; 1991: 159).

Drawing heavily on Schutz, Luckmann (1979: 199, 201; 1991: 159; 1983a [1973]: 28ff.) claims that phenomenology should serve as the basal proto-theory of sociological research. Thus, while the proto-language of the natural sciences is provided by mathematics and physics, the one adequate to sociology is of eminently *philosophical* nature. The social sciences in general and sociology in particular need a “philosophical foundation,” which they can only find in phenomenology (Luckmann 1979: 199).

Insofar as phenomenological analysis describes and conceptualizes the universal structures of (inter)subjective, meaningful experience, it offers a “rational and systematic understanding” of the fundamental features of sociology’s subject matter: pre-scientific social reality (Luckmann 1979: 199). To use Luckmann’s own terms, phenomenology provides a proto-language for adequately grasping, circumscribing, and defining the “field” of the social and its “constitutive elements” or minimal “units,” namely, (inter)subjective experiences and actions (Luckmann 1979: 201; 1983a [1973]: 23, 26). Only with the help of phenomenology, is it possible to find well-founded answers to central methodological questions of interpretive social research such as the following: “How can the ‘meanings’ constitutive of action be recognized, described and reconstructed as ‘data’? What are the ‘units’ of meaning in view of the obvious circumstance that they cannot be identified and segregated physically and measured in a space–time manifold? (Luckmann 1989: 18).

In a nutshell: according to Luckmann (1983a [1973]: 25), lifeworldly phenomenology should operate as a “*mathesis universalis* of social reality”.^16^ It provides empirical social researchers with a universal “‘grammar’ of action (and orientation)” [*Handlungs- *(*und Orientierungs-*)*Grammatik*], as it were, that allows them to apprehend, classify, and compare concrete social phenomena in a meaning-adequate, accurate, and reliable manner (Luckmann 1991: 162). In this sense, it acts as a “general” interpretative “matrix,” or “framework,” “for describing human action” (1991: 165). To use Hans-Georg Soeffner’s (1999: 37) terms, the Luckmannian theory of the lifeworld constitutes a “proto-sociology,” insofar as it defines the “subject matter” [*Gegenstandsbereich*] of the social sciences.^17^

## Three Strengths of the Luckmannian Account

Before proceeding to my constructive criticism of Luckmann’s position, I would like to discuss what I consider to be its major merits. It is my contention that his programmatic account has three major strengths. First (1), it provides a concise, clear, and operationalizable model for applying phenomenological insights in sociology. Second (2), it makes the effort to establish a bond between both disciplines without neglecting the specificities of the phenomenological method. And third (3), it tries to remain faithful to Schutz’s original theoretical project.

### An Operationalizable and Workable Model

Although Luckmann does not conceive of phenomenological analysis as an empirical research method, his programmatic account provides interpretive sociologists with an operationalizable model for making phenomenological insights fruitful in their research praxis. Understood as a proto-theory for sociology, phenomenology offers researchers a general conceptual heuristic for reconstructing concrete actions, experiences, and “small social life-worlds” (see Honer 2011: 22) in a reliable, accurate, and meaning-adequate manner. To put it differently, with the help of phenomenologically gained categories, such as those concerning intersubjectivity and lived spatiotemporality, empirical sociologists are able to enhance their *theoretical sensitivity* for seeing, describing, understanding, and interpreting the rich experiential-meaningful texture of empirical socio-cultural phenomena.

Furthermore, as Luckmann (1979: 204; in Dreher and Göttlich 2016: 34) suggests, phenomenology *qua* proto-sociology can serve as a solid theoretical framework for systematically comparing results of different empirical researches. Indeed, using the theory of the lifeworld as a “*tertium comparationis,*” sociologists can analyze the similarities and differences between the social construction of reality of different cultures (Luckmann 1979: 204). Such a comparison should be organized alongside the formal categories which, according to phenomenology, describe the invariant structures of human experience (Luckmann in Dreher and Göttlich 2016: 34).

The Luckmannian way of “applying” phenomenology in empirical social research in terms of a “proto-sociology” has especially shown its usefulness and productivity in the broad field of German-speaking sociology of knowledge. In this respect, empirically oriented approaches such as Hitzler and Honer’s (see 2015) “Life-World-Analytical Ethnography” [*Lebensweltanalytische Ethnographie*], Schnettler and Knoblauch’s “Ethnophenomenology” [*Ethnophänomenologie*] (Schnettler 2008), and Dreher’s (2012) “applied phenomenology” [*Angewandte Phänomenologie*] are worthy of mention (see Eberle 2010: 134ff.; 2021b; forthcoming; Eberle and Schnettler 2021; forthcoming).^18^ In spite of their undeniable differences, all these approaches conceive of phenomenological analysis not as an empirical method comparable to interviews or ethnographic observation but, rather, as a theoretical foundation for qualitative research. In this sense, they prefer speaking of “phenomenology-based” or “phenomenologically oriented” sociology instead of “phenomenological sociology” (see, for instance, Raab et al. 2008; Eberle and Schnettler 2021; forthcoming).^19^

### Respecting the Specificities of the Phenomenological Approach

Another strength of the Luckmannian approach lies in its effort to establish a cooperation between phenomenology and interpretive sociology without disregarding the specificities of phenomenological research. By contrast, it seems to me that the use of the hybrid label “phenomenological sociology” sometimes ends up occluding the peculiarity of phenomenology as a method and an intellectual tradition (see Luckmann 1979: 196; Raab et al. 2008: 11). The phenomenological approach is incorporated, or even mixed in, into empirical social researches, as it were, but this too often comes with the price of neglecting some of its fundamental traits. In this sense, as paradoxical as it sounds, phenomenological sociology is at times not very phenomenological (see Psathas 1989: IXff.; 1973: 14).^20^

The *eidetic* character of phenomenological reflection is one of those central features of phenomenology sometimes overlooked by “phenomenological sociologists”. As Luckmann rightly stresses, phenomenological philosophy, as classically developed by Husserl (Hua III: § 9) and his disciples (see, for instance, Merleau-Ponty 1945: I), aims at describing the *universal* structures of life-worldly experience. Indeed, as opposed to empirical interpretive sociology, when investigating social reality phenomenologists do not focus on the *haecceitas* of a concrete socio-historical phenomenon. Rather, using the method of eidetic variation, they seek to develop a “regional ontology” of sociality (see Husserl Hua III: § 9).

Luckmann’s attempt to connect phenomenology with sociology never loses sight of this and other fundamental traits of the former, such as the centrality of the first-person perspective, the use of a reflective, reductive, and descriptive methodology, and the pivotal role of the notion of constitution.

### Faithfulness to Alfred Schutz’s Thought

In this connection, it is also significant that Luckmann’s approach tries to remain faithful to Schutz’s work, i.e., to offer an “*exact exegesis*” thereof (Eberle 1993: 295) and to further develop it in a consistent manner (Luckmann in Dreher and Göttlich 2016: 31).^21^ To use Soeffner’s (1999: 29) words, in *Strukturen der Lebenswelt*, “Luckmann’s concern was to reconstruct his [Schutz’s] thought as ‘faithful’ as possible, without denying his own ideas”. To be sure, this faithfulness is not a *systematic* strength in itself; rather, it constitutes a merit in *intellectual-historical* terms, especially over those approaches that claim to follow Schutz but deviate partially or totally from his original position.

It seems to me that Luckmann’s thesis that the phenomenology of the lifeworld should not be seen as an empirical social research method but, rather, as a proto-theoretical foundation for sociology is perfectly in line with the Schutzian approach. As Schutz writes in a 1945 paper:

It must be clearly stated that the relation of phenomenology to the social sciences cannot be demonstrated by analyzing concrete problems of sociology or economics, such as social adjustment or theory of international trade, with phenomenological methods. It is my conviction, however, that future studies of the methods of the social sciences and their fundamental notions will of necessity lead to issues belonging to the domain of phenomenological research (Schutz 1962b [1945]).

Indeed, as many experts agree (Eberle 1993: 297; Embree 2017), the main aim of Schutz’s work consists in providing a solid epistemological foundation for interpretive sociology by developing a phenomenological “theory of the lifeworld” (Endreß 2006: 340). More precisely, Schutzian phenomenology intends to define and conceptually articulate the essential features of the subject matter of sociology, namely, pre-scientific social reality, and to determine the methodological postulates to be followed in its study (Embree 2017; see Schutz 1962a [1953]). With this in view, it becomes understandable why Luckmann (in Dreher and Göttlich, 2016: 31) maintains that Schutz was not a sociologist in the strict sense of the term but, rather, “the first practitioner of proto-sociology”.

## A Constructive Critical Assessment of the Shortcomings of Luckmann’s Position

Luckmann’s clear-cut differentiation between phenomenology and sociology is instructive and methodologically useful, insofar as it reduces the extreme complexity implied in the relationship between both research fields. However, just like every other schematic conceptual distinction, this one also has its disadvantages: if not nuanced properly, it can lead to dangerous theoretical imprecisions. By means of constructive criticism, in what follows I will attempt to refine some of the shortcomings of the Luckmannian account. My argument, which is informed by contemporary approaches in sociological theory and phenomenology, will center around three important issues: (1) Luckmann’s account of sociology, (2) his conception of phenomenology, and (3) his (in)sensitiveness with respect to the limits of the phenomenological reductions.

### Sociological Theory, Social Theory, and Theory of Society

In some of the passages in which Luckmann (see, for instance, 1979: 196, 200) deals with the relationship between sociology and phenomenology, he uses the concept of “sociology” in a somewhat inaccurate manner—for instance, when he claims that there is no such thing as a “phenomenological sociology” (1979: 196). For, as every sociologist knows, the word “sociology” on its own is too unspecific. Arguably, it could mean at least three different things: *qualitative empirical sociology*, *quantitative empirical sociology* or *sociological theory.*

As said above, an exhaustive analysis of Luckmann’s writings shows that he tends to equate sociology *in toto* with Weberian *Verstehende Soziologie*, that is, with a qualitative strain of empirical social research. If this is what he means by the term “sociology” in his programmatic writings, then his claim that a “phenomenological sociology” is a “conceptual contradiction” (Luckmann 1979: 196) can be simply understood as a criticism of Psathas’s (1973, 1989) attempt to use phenomenology as an empirical method. Interpreted this way, Luckmann’s statement seems plausible and well-founded.

Now, what is Luckmann’s take on *sociological theory*? Is it for him also sociology? Or, put differently, does Luckmann’s claim as to the impossibility of an *empirical* phenomenological sociology also hold for a *theoretical* phenomenological sociology? In my view, Luckmann does not provide clear answers to these questions.

It is easy to prove that sociological theory, i.e., the theoretical tradition whose founding fathers are Weber, Durkheim, Marx, and Simmel (see Kottmann et al. 2007), plays a pivotal role in Luckmann’s thought. And this especially in light of his most popular book, *The Social Construction of Reality*, which he and his co-author characterize as a work on systematic “sociological theory” (Berger and Luckmann 1966: 18). Surprisingly enough, however, in his programmatic writings Luckmann (see, for instance, 1979; 2008) does not even mention the latter term: he only refers to empirical social research and phenomenological proto-sociology.

Indeed, in these texts proto-sociology seems to take on one of the traditional functions of sociological theory, namely, that of conceptually defining the “field” of the social. Now, what is exactly the relationship between the phenomenological, or proto-sociological, enterprise and the sociological-theoretical one? Can proto-sociology be understood as a sociological theory? Or is the idea of a “phenomenological sociological theory” also an absurdity?

In order to answer these questions, it is necessary to work with a thorough definition of the term “sociological theory,” which, unfortunately, is not to be found in Luckmann. In the last few years, especially in the German-speaking world, one observes a series of attempts to offer such a definition by means of introducing an interesting conceptual distinction, namely, that between *social theory* [*Sozialtheorie*] and *theory of society* [*Gesellschaftstheorie*] (see, for instance, Reckwitz 2016: 7f.). According to authors such as Andreas Reckwitz (2016: 7f.), sociological theory deals, since its origins, with two parallel problems that can and must be differentiated, namely, with *social-theoretical* [*sozialtheoretisch*] issues and *societal-theoretical* [*gesellschaftstheoretisch*] ones.

On the one hand, sociological theory *qua social theory* intends to conceptually define the fundamental features of the subject matter of sociology, namely, social reality, i.e., its necessary, transhistorical, and transcultural proprieties. In other words, social theory aims at defining *what is sociality* in terms of a social ontology, and this by developing a “fundamental vocabulary” of basal concepts for describing and analyzing social phenomena (Reckwitz 2016: 7). By contrast, sociological theory *qua theory of society* attempts to analyze the structure, historical development, and contemporary situation of *modern societies* or *Gesellschaften*, that is, of the capitalistic, industrial, rationalized, urbanized, democratic, and individualistic social formations that started emerging in Europe in the nineteenth century (Reckwitz 2016: 8).

Arguably, against the background of this distinction, Luckmann’s proto-sociology can be understood as a *phenomenological social theory*, for it seeks to offer a meta-language able to grasp the basal structures of social reality (see Schnettler 2008: 142).^22^ Interestingly enough, however, in *The Social Construction of Reality* Luckmann and Berger (1966: 20. My emphasis) do not consider the phenomenological reflections included in the first chapter of the book as a constituent part of their sociological theory but, rather, as “*pre-sociological*” or “*philosophical* prolegomena”. In this view, phenomenology is not a social-theoretical account per se but *precedes* social theory as its philosophical foundation.

Now, how does Luckmann conceive of sociological theory? “The central question for sociological theory,” one reads in *The Social Construction of Reality*, “can then be put as follows: How is it possible that subjective meanings *become* objective facticities? How is it possible that human activity (*Handeln*) should produce a world of things (*choses*)?” (Berger and Luckmann 1966: 18). According to Luckmann and Berger, this question can only be answered by a synthetic approach that combines Weber’s subject-centered account with Durkheim’s object-centered one, and this with the help of the young Marx’s dialectical conception of social reality (1966: 185f.).

Curiously enough, however, in his programmatic writings dealing with the relation between sociology and phenomenology, Luckmann (see, for instance, 2002a [1990]: 51) appears to contradict the division of labor between phenomenological proto- or “pre-sociology” (Berger and Luckmann, 1966: 20) and sociological theory presented in *The Social Construction of Reality*. In articles such as “*Lebenswelt: Modebegriff oder Forschungsprogramm?,*” he seems to suggest that the above-mentioned “fundamental problem” of sociological theory can only be tackled through a *philosophical-phenomenological* “foundation” of the social sciences (Luckmann 2002a [1990]: 51). As he writes, only phenomenological analysis is able “to clarify the relationship between the universal structures of subjective orientation, the fundamental forms of intersubjective action, and the objective features of historical social reality” (2002a [1990]: 51).

From this follows that Luckmann acknowledges phenomenology’s contributions to social theory. However, he does not specify the nature of such contributions, and this because he does not accurately define the boundaries between phenomenological proto-sociology and social theory *stricto sensu*. Does the former provide a philosophical foundation for the latter, as it is suggested in *The Social Construction of Reality*? Or is phenomenological proto-sociology already a social theory, as one can deduce from Luckmann’s programmatic writings?

Following Reckwitz (2016: 8), I would like to argue that Luckmann’s lack of precision when it comes to specifying the boundaries between (phenomenological) philosophy and social theoretical thought is not an exclusive flaw of his work. Rather, it reflects the inseparable bond that exists between both intellectual enterprises. Indeed, it is hard to say when philosophy ends and social theory begins, for the questions dealt with by the former, namely, the nature of human existence, human knowledge, social relationships, language, etc., also play a key role in the latter.^23^

### Transcendental or Pragmatic-Anthropological Phenomenology?

Arguably, Luckmann is also ambiguous when it comes to defining his understanding of phenomenology. He seems to oscillate between an orthodox account of phenomenological philosophy, which reminds one of Husserl’s static transcendental approach in *Ideen I* (see Belvedere 2013: 10), and a pragmatic, philosophical-anthropological, and social-theoretically oriented account decisively inspired by Schutz. While the former seems to be prevalent in the programmatic texts analyzed above, the latter can be found in his concrete analyses of the structures of the lifeworld and in some papers from the 1990s (see, for instance, Luckmann 2002a [1990]).

Luckmann’s portrait of phenomenology in programmatic writings such as “Phänomenologie und Soziologie” (1979) and “Konstitution, Konstruktion: Phänomenologie, Sozialwissenschaft” (2008) seems to reproduce, to some degree, the solipsistic, idealist, and cognitivist features of Husserl’s early conception of transcendental phenomenology (Belvedere 2013: 10; see Hua III). In these writings, Luckmann (1979: 196f.; see 2008: 33ff.) characterizes phenomenology exclusively as a purely egological philosophy that intends to work out the “universal structures of consciousness” by means of an exhaustive reflection upon the “inner stream of experience”. On this account, phenomenological research aims at describing the way in which the lifeworld “constitutes” itself in, and through, the cognitive-intentional “conscious activities” of the ego (Luckmann 2008: 33ff.). “Reality,” writes Luckmann in a Husserlian vein, “constitutes itself in conscious activities that intentionally grasp something outside consciousness itself” (34).

It seems to me that the solipsistic and mentalist connotations of this account are paradigmatically reflected in Luckmann’s dichotomy between phenomenological “constitution” and sociological “construction”. While he uses the term “construction” to denote those social processes in which human beings practically produce a socio-cultural world through corporeal-material (inter)actions (Luckmann 2008: 35; 2007b [2001]: 140), he tends to characterize “constitution” as a mere cognitive process of sense-giving occurring, as it were, within the pure interiority of a disembodied egological mind (Luckmann 2008: 34; 2007b [2001]: 140).

Arguably, as a result of this clear-cut conceptual distinction, it arises, against Luckmann’s own intentions, a one-sided picture of phenomenology as an outdated, Cartesian approach. And this, in turn, can create the impression that it has nothing whatsoever to offer sociology. Many contemporary authors rightly argue against such unilateral definitions of phenomenological philosophy, which seem to be prevalent in the contemporary social sciences (see, for instance, Belvedere 2011). As Dan Zahavi has recently written:

By offering an account of human existence, where the subject is understood as an embodied and socially and culturally embedded being-in-the-world, phenomenology is not only able to analyse and illuminate a framework that is operative and taken for granted by most scientific disciplines, it has also been able to offer inputs to a variety of disciplines in the social sciences and the humanities, including anthropology, sociology, psychology, literary studies, education studies, etc. (Zahavi 2018: 103f.)

Indeed, Luckmann’s definition of phenomenology in his programmatic texts seems to disregard that the most prominent representatives of this philosophical tradition, Heidegger (2006 [1927]), Merleau-Ponty (1945), Schutz (1962b [1945]), and even Husserl in his later writings (see Zahavi 2003: 98ff.), do not conceive of the constitution of the lifeworld as a purely cognitive activity taking place within the interiority of subjective mind but, rather, as an embodied and socio-culturally embedded process. That is to say, that the boundaries between “construction” and “constitution” are not as sharp as Luckmann sometimes depicts them.

Luckmann’s “orthodox” account of phenomenology becomes also manifest in his claim that phenomenological research necessarily operates with the method of transcendental *epoché*. In line with the Husserlian account of transcendental phenomenology, he claims that phenomenology can never be “naively realist” (Luckmann 1979: 197; 2007b [2001]: 140f.). It must suspend reality’s “ontological claims,” so to speak, for only that way is it possible to render visible how reality constitutes itself in our cognitive conscious acts (Luckmann 2008: 33).

In my view, this way of depicting phenomenology collides with Luckmann’s own *concrete* analyses of the structures of the lifeworld, which, as is well-known, follow Schutz’s (see 1991 [1932]: 55ff.) “constitutive phenomenology of the natural attitude,” and not Husserlian transcendental phenomenology (see, for instance, Luckmann 1992).^24^ Indeed, in his concrete phenomenological descriptions he portraits the human constitution of reality as an essentially embodied, socio-culturally embedded, and pragmatically shaped process. Concerning the corporeal nature of human action, for instance, he writes: “To be sure, the body is presupposed in every human action and in every experience, also in those actions that, as such, are not necessarily embodied” (Luckmann 2002b [1982]: 98).

In the 1973 paper “Philosophy, Sciences, and Everyday Life,” Luckmann (1983a [1973]: 21) clearly distances himself from the Husserlian transcendental approach. More precisely, he criticizes transcendental phenomenology’s “illusory quest for absolute and total certainty” and objects to the metaphysical idea of a “first philosophy” as “supreme ‘discipline of disciplines’” (Luckmann 1983a [1973]: 21f.). In a Schutzian vein, he suggests that one can pursue Husserl’s (Hua VI: § 36) program of an eidetic science of the lifeworld, as presented in *Krisis,* without accepting transcendental phenomenology (Luckmann 1983a [1973]: 21f., 28).

In his 1990 article “*Lebenswelt: Modebegriff oder Forschungsprogramm?,*” in turn, Luckmann admits again that he does not practice Husserlian transcendental phenomenology but, rather, Schutzian *mundane phenomenology* (Luckmann 2002a [1990]: 51; see also 1993). In line with his teacher, Luckmann claims that his phenomenological proto-sociology does not aim at offering a “dogmatic-definitive ultimate foundation” for philosophical knowledge but, rather, a “philosophical foundation” for sociology *qua* “empirical science” (2002a [1990]: 50f.). As he writes, the “Schutzian turn” of phenomenology has helped to reveal the “important function” of this discipline for the social sciences (2002a [1990]: 50).

When characterizing the specificities of the Schutzian approach, Luckmann follows Ilja Srubar’s (2007) pragmatic and philosophical-anthropological reading of Schutz’s work*.* Srubar (2007: 173ff.) rightly emphasizes that Schutz progressively distances himself from Husserl’s transcendental approach to phenomenology, and this because of its –alleged– metaphysical, idealist, and solipsistic features. On this reading, as opposed to his teacher, Schutz develops a phenomenology of the natural attitude which aims to be an “ontology of the lifeworld” (176). This approach, says Srubar, implies not only a *philosophical-anthropological* and *pragmatic* “turn” of phenomenology but also its “*sociologization*” (18. My emphasis).

Interestingly enough, Srubar (2007: 18, 68) does not operate with Luckmann’s dichotomy between sociological construction and phenomenological constitution. Rather, he opts for using the term “social constitution” [*soziale Konstitution*] in a broad sense to designate *all* the processes and mechanisms through which the lifeworld obtains its “meaning-structure” and “-order” (2007: 23, 91). Against this background, he argues that both Husserl and Schutz provide theories of social constitution, although they differ as to their respective definitions of the “lifeworldly constitution processes,” and this, ultimately, because they pursue different theoretical aims (2007: 18, 68).

From an “epistemological” and “metaphysically” connoted perspective, suggests Srubar (2007: 17f., 117), Husserl assumes that the meaning structure of the lifeworld ultimately constitutes itself in the cognitive activities of an absolute transcendental consciousness.^25^ By contrast, arguing from an “ontological” and “sociologically” oriented perspective, Schutz understands the constitution of the lifeworld as a *pragmatic, embodied, and intersubjective* process taking place in everyday socio-cultural praxis (18f., 68, 188).

More precisely, according to Srubar (2007: 17, 68, 189f., 198), Husserl posits the internal stream of consciousness of the transcendental ego as the primary “location” [*Ort*] of the constitution process of the lifeworld, while Schutz conceives the face-to-face intersubjective interaction, the so-called “*Wirkensbeziehung,*” as the “primal cell” [*Urzelle*] of social reality. In Srubar’s reading, however, Schutz’s claim of the ontological primacy of intersubjectivity over subjectivity does not imply reducing egological consciousness to a mere epiphenomenon of sociality. Just like in Husserl, in Schutz subjective “perspectivity” and “meaning-giving” also play a crucial role in the constitution of reality (Srubar 2007: 181, 191).

As Srubar (2007: 23, 191) claims, for Schutz the lifeworld obtains its “meaning-structure” from two co-originary [*gleichursprünglich*] “constitutive poles,” namely, an *intersubjective,* or *social*, and a *subjective,* or *egological,* one (see Eberle, 1993: 304). As a result of intersubjective interaction, it emerges a socio-cultural world with an own “relevance and typicality structure,” i.e., a substantive, objective reality that not only transcends the interacting subjects and their respective intentions but also is imposed upon them (Srubar 2007: 23, 191). In turn, the egological constitutive pole of Schutz’s theory of the lifeworld consists in the perspective “appropriation” and “modification” of that imposed social reality performed by each individual within the frame of her personal “biography” (Srubar 2007: 23).

Explicitly following Srubar’s reading, Luckmann (2002a [1990]: 49f.; 1993) speaks in some of his writings from the 1990s of a “Schutzian turn” in phenomenology. Schutz, he says, distances himself from Husserl’s transcendental approach and develops a “phenomenology of the lifeworld” which is “pragmatically-anthropologically founded” (Luckmann 2002a [1990]: 50). But this is not all. Although he uses a different terminology, Luckmann also seems to accept Srubar’s claim that Schutz’s theory of the lifeworld possesses two constitutive poles: a subjective and an intersubjective one (2002a [1990]: 49f.). As he writes, “in the analysis of the ‘meaningful construction [*Aufbau*] of the social world’, one has to reconstruct both the constitution of *meaning* [*Sinn*] in subjective conscious activities […] and the *construction* [*Aufbau*] of reality performed in social action” (2002a [1990]: 50).

In his own work, Luckmann accepts and takes the Schutzian anthropological turn of phenomenology further (Soeffner 1999: 36). Indeed, he argues that the eidetic structures of the lifeworld are anthropological invariants, i.e., fundamental aspects of the “*conditio humana*” (Luckmann in Dreher and Göttlich 2016: 33), and, based on this assumption, develops a phenomenologically *and* philosophical-anthropologically founded proto-sociology that complements Husserl’s and Schutz’s reflections with anthropological insights from the young Marx, Helmuth Plessner, and Arnold Gehlen (Berger and Luckmann 1966: 17; see Schnettler 2006: 69, 90).

### The Limits of the Phenomenological Reductions

Arguably, some of Luckmann’s programmatic formulations convey the impression that he overestimates the powers of the phenomenological reductions. He seems to depict them as infallible methodological devices that allow us to completely detach ourselves from all biographical, cultural, and historical prejudices, as it were, thereby enabling us to see the “things themselves,” i.e., the universal structures of the lifeworld, *sub specie aeternitatis* (see Eberle 2012b: 144; Göttlich 2008: 103ff.)*.* Understood this way, phenomenology would make it possible to develop a foolproof proto-sociological, or social theoretical, language able to overcome all contingent biases and sources of error when it comes to defining the general and fundamental concepts of sociology.

As Luckmann (for instance, 1979: 198; 2008: 38; 1983b [1970]: 41) writes on many occasions, through the application of both the *epoché* and the eidetic variation, it is possible to reduce a particular, concrete social phenomenon to its most elementary features, i.e., to those invariant structures of intentionality without which such kind of experience or action would be unthinkable. On this account, with the help of the phenomenological reductions one is able to strictly differentiate the “fundamental strata” of experience from its secondary –biographical, socio-cultural, and historical– constituent elements, which are necessarily based on the former (Luckmann 2007b [2001]: 141).

Now, in line with Eberle (2012b: 144; 2021b; forthcoming), and arguing from a post-metaphysically (see Habermas 1988: 36ff.) and hermeneutically informed perspective that emphasizes the phenomenologist’s finitude and her insurmountable “belonging-to” the lifeworld she reflects upon (see Ricouer 1975), one can ask: Is it truly possible to separate so neatly the factual from the essential moments of a concrete experience? Can a phenomenologist completely detach herself from her particular situation and observe the universal structures of the lifeworld from the perspective of the eternal? Is it really viable to consider *all the possible variations* of experience only by means of an egological reflection? Or does a really exhaustive eidetic variation require us to resort to the results of empirical social research?^26^

In some passages of his work, Luckmann acknowledges, at least *en passant*, the insurmountable limitations of phenomenological reflection, suggesting that the reductions are far from being infallible. Concerning the problem of language, he claims in a 1991 paper that phenomenological analysis only brackets “*as best as possible*” the theoretical and pre-theoretical prejudices of common-sense thinking, which means that the phenomenological proto-language always retains some “traces” of the contingent historical languages (Luckmann 1991: 157. My emphasis). And in another article from 1970, he admits that

it is extraordinarily difficult to separate the “pure” qualities, appearing in the primordial sphere of the transcendental ego, from the culturally determined categories which are first acquired by the empirical ego in social processes and then recede into “secondary passivity” and thus guide its habitual experiences. (Luckmann 1983b [1970]: 46)

In Luckmann, however, one does not find a systematic reflection on the insurmountable limitations of the reductions when it comes to separating historical-cultural facticity from the (transcendental-)eidetic realm. In this respect, arguably, Schutz (1970 [1959]: 115) goes further than his disciple when he asks himself, in a 1959 paper, if the eidetic variation can ever surpass the limits of the typifications that organize the contingent common-sense experience of the phenomenologist. It is certainly Merleau-Ponty (2010 [1951–1952]), however, who most thoroughly deals with this problem, especially in his course “*Les sciences de l’homme et la phénoménologie*”. In the following, I will attempt to apply Merleau-Ponty’s insights on this matter to a critical discussion of Luckmann’s position.

Merleau-Ponty (2010 [1951–1952]: 1211ff.) develops his own account of the scope and limits of the phenomenological reductions in an *indirect* manner, namely, through an exegesis of Husserl’s work. More precisely, he criticizes (1) a “dogmatic” conception of the reductive methods that disregards their fallibility and limitations, and defends instead (2) a more modest account that takes their insurmountable historical and cultural embeddedness seriously. The first position, claims Merleau-Ponty, is to be found in the “first” Husserl, while the second can be identified in his later work (2010 [1951–1952]: 1211ff.). Arguably, Luckmann’s programmatic formulations regarding the relation between phenomenology and sociology rest on an account of the reductions of the first type (1). In this sense, I think Merleau-Ponty’s defense of the second kind of approach (2) can serve as a corrective for the Luckmannian position.

(1) The first account, which, according to Merleau-Ponty (2010 [1951–1952]: 1211, 1213), is endorsed by Husserl in texts such as *Ideen I*, starts from the premise that, by means of the *epoché* and the eidetic variation, one is able to detach herself completely from one’s socio-historical situation, thereby being able to observe the universal structures of the lifeworld from an Archimedean point. In this view, for instance, performing the reduction enables the phenomenologist to entirely transcend the limitations of her mother tongue and thus to analyze, from a completely unbiased vantage point, the invariant “essence of language” in terms of a “universal grammar” (2010 [1951–1952]: 1245f.).

As Merleau-Ponty (2010 [1951–1952]: 1222) rightly suggests, this conception of the reductive methods goes hand in hand with a dogmatic account of the phenomenological “essences,” which tends to underestimate the relevance of both concrete socio-historical facticity and the social scientific efforts to study it. On this account, phenomenologists deal with the “essential,” i.e., with the necessary and invariant structures of social reality, while social scientists only occupy themselves with factual “details” that are nothing but empirical substantiations thereof (2010 [1951–1952]: 1231). To use Husserl’s (Hua III: §§ 7, 8, 9) terms in *Ideen I*, the “factual” social sciences *qua* “*Tatsachenwissenschaften,*” i.e., empirical sociology, anthropology, linguistics, etc., are always subordinated to the dominance of the “eidetic sciences” [*eidetische Wissenschaften*] (Merleau-Ponty 2010 [1951–1952]: 1222f., 1230). If one follows this view, phenomenology defines a priori the “fundamental concepts” of the empirical social sciences, while the latter limit themselves to collecting factual “curiosities” and to making sense of them in light of those eidetic categories (2010 [1951–1952]: 1231).

(2) By contrast, the second account of the reductions, which Merleau-Ponty (2010 [1951–1952]: 1257) identifies in Husserl’s later writings, acknowledges the inevitable naivety and fallibility of the phenomenological method. In this view, a phenomenological reflection is not “radical” when it overcomes historicity and facticity once and for all. In fact, such a thing is not possible. Rather, a radical reflection is one that accepts as a starting point its insurmountable embeddedness in a historical situation, which is always already lived unreflectively: “radical reflection amounts to a consciousness of its own dependence [*dépendance*] on an unreflective life which is its initial situation, unchanging, given once and for all” (Merleau-Ponty 2002 [1945]: xvi; 1945; ix).

On this account, the *epoché* is far from being an infallible method which succeeds at completely bracketing our historically determined prejudices and cognitive biases, thereby allowing us to “come back” to the primal transparence of the transcendental sphere (Merleau-Ponty 2002 [1945]: x; 1945: v). Rather, it constitutes an “*infinite*” task, i.e., an imperfect and never completed “effort” to take some distance from our factual, unreflective situation in order to “contemplate” it, articulate it, and thus make it understandable (Merleau-Ponty 2010 [1951–1952]: 1211). In other words, doing away with our embeddedness in a concrete socio-historical lifeworld by means of phenomenological reflection is simply not an option: “The most important lesson which the reduction teaches us is the impossibility of a complete reduction” (Merleau-Ponty 2002 [1945]: xv; 1945: ix).

Something similar holds for the eidetic reduction. Merleau-Ponty (2010 [1951–1952]: 1246, 1255, 1263f.) criticizes the platonic-sounding position of the “first” Husserl and defends a more modest understanding of this method, which he finds in the work of the later Husserl. On this latter account, he says, the intuition of essences or *Wesensschau* is not an “end in itself” but, rather, a modest methodological “means” to conceptually fix, so to speak, the fundamental features of our lived experience (Merleau-Ponty 2002, XVIf.; 1945: VIIIf.). And far from providing us with an apodictic knowledge of the essential structures of reality, it always entails a “certain degree of naivety,” which follows from the insurmountable historical and cultural limitedness of human thinking (Merleau-Ponty 2010 [1951–1952]: 1264).

If one takes the socio-historical limitations and biases of cognition seriously, then it seems absurd to claim that one can consider *all possibilities of experience* by means of a methodical reflection. Indeed, if the scope of our cogitations is always already narrowed because of our embeddedness in a particular situation, then we are not able to ponder *all* the possible variations of a phenomenon in a single act of eidetic variation. Take, for instance, a phenomenologist who looks back on a *Wesensschau* she performed twenty years ago: she may find out that the range of her imaginary variation was restricted by the stock of knowledge socially prevalent back then and, therefore, that she was not really in presence of the essence of things themselves (see Merleau-Ponty 2010 [1951–1952]: 1263).

## Final Words: Towards a “Dialogical” Parallel Action

The account of the relation between phenomenology and sociology as a “parallel action” [*Parallelaktion*], presented by Luckmann in some of his texts (1979: 197; 2007c [1999]: 131) and further developed especially by Jochen Dreher (2012), has an important merit: in contrast to the synthetic or hybrid approach of “phenomenological sociology,” prominent in the US context, it allows for establishing a linkage between both disciplines without disregarding their respective specificities. Broadly speaking, the idea of *Parrallelaktion* refers to the combined application of two different procedures, namely, phenomenological analysis, on the one hand, and the methods of qualitative sociology, on the other, in the study of social phenomena (see Eberle 2021b; forthcoming; Dreher 2012). More precisely, according to this approach, the two disciplines should behave like parallel lines, meaning, they should run in the same direction but never intersect or mix with each other. Each of them has to do its job separately, as it were, and thereby contribute to the common effort of investigating social reality (or a segment thereof).^27^

I think the metaphor of parallelism is quite useful for emphasizing the importance of not forgetting the discrepancies between phenomenology and empirical social research when bringing them into relation with each other. Indeed, if one *seriously* wants to reflect on the contributions of the former to the latter, or vice versa, then one is obliged to respect their respective identities. Otherwise, one is not establishing a relationship between them but, rather, conflating and fusing them into an artificial construct.

However, as scholars such as Göttlich (2008: 103) and Eberle (2021b; forthcoming) rightly suggest, Luckmann’s use of the metaphor of parallelity also has its disadvantages.^28^ While it can account for, and do justice to, the divergences between both disciplines, it seems to be unable to connote their points of convergence, i.e., the way(s) in which they could cooperate with each other. Does it make sense to speak of a collaboration or interaction between two *parallel* lines of research, that is, between two levels of thought that go in the same direction but *never meet*? In my view, the rather abstract idea that parallel lines do intersect at infinity (see Wikipedia 2021a; 2021b), sometimes used by advocates of the *Parallelaktion* to counteract this objection, does not help much in this respect.

The inability of the metaphor of parallelism for denoting the cooperation between phenomenology and empirical sociology becomes especially problematic if one, as I do, accepts Merleau-Ponty’s analysis of the limits of the phenomenological reductions. From a Merleau-Pontyan perspective, the relationship between both disciplines should not be one of strict separation and/or asymmetrical subordination but, rather, one of “reciprocity,” “intertwining” [*entrelacement*] or, to use a term by Gallagher and Zahavi (2008: 220), of “mutual enlightenment”. In this view, not only phenomenology can contribute to theoretically underpinning empirical social research; the latter can also help phenomenologists overcome, at least partially, the historical, cultural, and biographical limitations of their eidetic reflections (Merleau-Ponty 2010 [1951–1952]: 1261).^29^

It seems to me that a good way of correcting the stated shortcoming of the notion of *Parallelaktion* without losing its strengths is by combining it with another metaphor, namely, that of “dialogue”. To put it more precisely, I would like to argue that the relationship between phenomenology and sociology should be understood as a *dialogical parallel action*, that is, as the simultaneous action of two *parallel* methodological approaches that are in constant *dialogue* with each other. Seen this way, the two parallel lines (of research) run independently from each other but, at the same time, are engaged in a reciprocal conversation, which, importantly, does not undermine their respective identities.

The concept of dialogue implied in my idea of a *dialogical Parallelaktion* is inspired by Hartmut Rosa’s (2016: 285, 295, 298) theory of resonance. According to Rosa, a true, “responsive” or “resonant,” dialogue between two interlocutors does not entail a complete fusion of their horizons into an undifferentiated unity. Rather, it should be seen as a “conversation” between “two more or less discrete entities,” each of which “hears” and “responds” to the other with its “own voice” (286, 295, 298). This way of understanding dialogue is compatible with the metaphor of parallelism because it assumes that both poles of the dialogical relation must be “open enough” to listen to, and to be irritated by, their counterpart but also remain “closed enough” to not lose their respective idiosyncrasies in the process (298).

In my view, in perhaps the most relevant paper in which Luckmann (1983b [1970]) puts its programmatic account of the relation between phenomenology and sociology into practice, “On the Boundaries of the Social World,” one finds implicit traces of this *dialogical Parallelaktion*.^30^ Broadly speaking, this 1970 article deals with an important (social) theoretical problem, namely, that of the definition of the boundaries between social and non-social reality, i.e., the question as to how societies define the demarcation limits between those beings or entities that are, both cognitively and morally, recognized as members of the social world and those which are not. In order to tackle this issue, Luckmann simultaneously resorts to phenomenological reflections and to a discussion of findings of empirical social research.

Following the parallel action approach, he sharply differentiates between both planes or forms of thinking. Phenomenological constitutive analysis is one thing; the qualitative reconstruction of historical social constructions is another (see Luckmann 1983b [1970]: 44, 46, 52f.). However, it seems to me that in this classical essay, the parallelism of both analytic procedures goes hand in hand with a rudimentary dialogue of mutual enlightenment between them, a dialogue which, unfortunately, is not explicitly identified by the author. On the one hand, Luckmann’s phenomenological analysis of the operation of consciousness responsible for the constitution of the boundaries of the social world, namely, the “universal projection,” appears to be mediated, at least implicitly, by empirical findings concerning the onto- and phylogenetic primacy of animism (see Luckmann 1983b [1970]: 42–47). And on the other hand, his theoretical interpretation and discussion of those findings are, to a great extent, phenomenologically informed (1983b [1970]: 47–61).^31^

Arguably, Luckmann was the first practitioner of the *dialogical parallel action.* And contemporary followers of his approach, such as Raab, Pfadenhauer, Stegmaier, Dreher & Schnettler (2008: 13), also seem to agree with this idea when, without renouncing to the metaphor of parallelism, they promote a “mutual information and irritation” between phenomenology and empirical sociology.

## Footnotes

1 For an exhaustive reconstruction of Psathas’s account, see Belvedere (2020). Eberle (2012a; 2014: 191f.) shows the crucial role played by Harold Garfinkel (see 1967) in the reception of Schutz in the US, emphasizing his influence on the work of Psathas. Although Psathas’s phenomenological sociological approach is different from Garfinkel’s ethnomethodology, it is in line with the latter’s attempt to apply phenomenology as a method of social research (Eberle 2012b: 135f.).

2 Endreß and Srubar (1997) give a thorough overview of the history of the reception of Schutz’s work in German-speaking sociology.

3 Luckmann’s popularity is especially due to his co-authorship of two important works: *Strukturen der Lebenswelt*, the completion of the posthumous work of his teacher, Alfred Schutz (Luckmann and Schutz 2003 [1979]), and *The Social Construction of Reality*, written in collaboration with Peter Berger (Berger and Luckmann 1966). However, he is also well-known worldwide for his writings on sociology of religion (Luckmann 1967).

4 By Luckmann’s “programmatic account,” a term I will use frequently in the present paper, I mean his systematic position on the issue of the relation between phenomenology and sociology as paradigmatically presented in texts such as “Phänomenologie und Soziologie” (Luckmann 1979), “Philosophy, Science and Everyday Life” (Luckmann 1983a [1973]), “Protosoziologie als Protopsychologie?” (Luckmann 1991), and “Wirklichkeiten: Individuelle Konstitution, gesellschaftliche Konstruktion” (Luckmann 2007c [1999]).

5 Although they do mention Luckmann and discuss some of the theses presented in *The Social Construction of Reality*, most of the recent English-speaking publications on so-called phenomenological sociology do not expound, let alone discuss, the Luckmannian programmatic account of the relationship between both disciplines (see Zahavi, 2018; Overgaard and Zahavi, 2009; Bird 2009). Something similar occurs in the Spanish-speaking world (see Rizo García 2015), except for a very few exceptions (see Belvedere 2013). In this sense, Eberle (2021b; forthcoming) claims that Luckmann’s account of the relationship between phenomenology and sociology is “completely ignored” outside the German-speaking context.

6 In the German-speaking literature (Schnettler 2006: 69ff.; Knoblauch et al. 2002), and also in some English-speaking texts written by German-speaking authors, one finds good presentations of the Luckmannian programmatic account and even interesting attempts to apply it in concrete social research (Dreher 2012). However, one does not encounter an exhaustive constructive criticism of it as the one Luckmann (1993: 321) himself expected. In this respect, Thomas Eberle’s (2012b: 144 f.; 2021b; forthcoming) critical remarks constitute an exception. In Belvedere (2013: 10) and Göttlich (2008: 103) one also finds some interesting objections to Luckmann’s position.

7 Luckmann takes the concept of “parallel action” from Robert Musil’s novel *Der Mann ohne Eigenschaften* (Dreher and Göttlich 2016: 34f.)*.*

8 For Luckmann (1978: 8, 11), phenomenology is egological insofar as it proceeds from a “personal,” first-person perspective, meaning, it takes “the individual human being as the centre of a system of coordinates on which the experience of the world is mapped”. Cosmological sciences, in contrast, adopt an “impersonal,” third-person perspective (11).

9 I use here the concept “a priori” to characterize the universal structures of the lifeworld, although Luckmann himself (in Dreher and Göttlich, 2016: 33) “do[es]n’t like the term”.

10 In line with Husserl (see Hua III: 77), by reflection Luckmann (1978: 8) means the “inspection of our own experiences “.

11 It is Luckmann (2008: 34; 2007c [1999]: 128) himself who repeatedly claims that intentional objects are “outside” consciousness. However, he does not explicate this claim further. He is probably referring to the fact that, for Husserl (Hua XIX 1: 385f.; Hua XVII: 148), the *noema* or *cogitatum* “transcends” the “real” [*reel*], immanent content of consciousness.

12 Luckmann (for instance, 2008: 35) does not distinguish between the “*epoché*” and the “reduction” but uses both terms synonymously just like Husserl himself does in several of his writings (for instance, Hua I: 10f.; Hua III: 73). Currently, Husserl scholars such as Dan Zahavi (2018: 37f.) insist on the difference between both notions, characterizing them as two distinct steps in transcendental-phenomenological reflection. The former denotes the suspension or bracketing of the natural attitude which opens the “gate of entry” to the transcendental realm, as it were (2018: 37); the latter, in turn, designates the “systematic analysis of the correlation between subjectivity and world,” or, more precisely put, the analytic reconduction (from the Latin verb “*re-ducere*”) of the world to its constitution in transcendental subjectivity (2018: 38; see Hua I: 21).

13 For a criticism of Luckmann’s account of measurement in sociology, see Eberle (2021b; forthcoming).

14 Luckmann’s concept of “proto-sociology” is inspired by Peter Janich’s conception of “proto-physics”. Janich uses the term to refer to the “philosophy of physics,” which is not physics per se but, rather, an analysis of the “presuppositions of *doing* physics” (Luckmann in Dreher and Göttlich 2016: 32; see Luckmann 1989: 19).

15 Importantly, Luckmann’s strong criticism of the state of the art of empirical sociological research is, in part, inspired by Aaron Cicourel’s (see 1964: iii ff.) approach in his 1964 book *Method and Measurement in Sociology* (Luckmann 1983a [1973]: 27, 36; 1979: 203). For an in-depth and critical discussion of Cicourel’s account and of the idea of a proto-sociology (see Habermas 1982: 212ff.).

16 It follows from what I have said so far that Luckmann (1983a [1973]: 28) uses the term “*mathesis universalis*” in a “restricted metaphorical sense”.

17 Soeffner (1999: 33) also characterizes Schutz and Luckmann’s theory of the lifeworld as a “proto-hermeneutics” [*Protohermeneutik*], insofar as it analyzes the a priori structures of pre-scientific and scientific “understanding” [*Verstehen*]. In this sense, he claims, phenomenology clarifies not only the subject matter but also the *method* of the social sciences. One finds the same characterization in Eberle (2010: 137; 2021b; forthcoming).

18 In this connection, Eberle’s approach of a “phenomenological hermeneutics” [*phänomenologische Hermeneutik*] is also worthy of mention (see Eberle 2021a; forthcoming; Eberle and Schnettler, 2021; forthcoming). Although he criticizes some aspects of Luckmann’s programmatic approach (see Eberle 2021b; forthcoming; 2012b: 144), Eberle (2021a; forthcoming) also conceives of phenomenological analysis essentially as a philosophical foundation of qualitative sociology, and not as an empirical research method per se.

19 Interestingly enough, prominent contemporary phenomenologists such as Dan Zahavi (2018) adopt a similar approach concerning the application of phenomenology to qualitative empirical research in the human sciences.

20 Psathas (1989: IXff.), for instance, provides a heterodox reading of phenomenology which, in my view, is at odds with some central features of this tradition of thought. On the one hand, he considers the theoretical developments of authors such as Mead, Weber, Cooley or Simmel as phenomenological contributions. (As Luckmann (1978: 10) rightly notes, “[n]either Max Weber nor Mead was a phenomenologist –*avant-(ou après)-la-lettre*”). And on the other hand, he understands “phenomenological sociology” as a “new paradigm” in empirical social research alternative to positivist sociolology (see Eberle 2014: 191f.). By saying this, of course, I am not denying the great value of Psathas’s work.

21 Eberle (1993: 295) maintains that, in general terms, the Luckmannian reading of Schutzian phenomenology as a proto-sociological enterprise is adequate. However, he criticizes Luckmann’s polemical decision of excluding from *Strukturen der Lebenswelt* Schutz’s final chapter on “methodology of the social sciences” (see Eberle, 2010: 124)*.* Other divergences between Luckmann and Schutz are pointed out and discussed in Eberle (2021b; forthcoming).

22 Schnettler (2008: 142) characterizes Luckmannian protosociology as a “phenomenologically founded social theory”.

23 Eberle (2021b; forthcoming) makes a similar point.

24 Luckmann neither refers to Husserl’s distinction between transcendental phenomenology and phenomenological psychology nor to its importance for Schutz’s thought. For an analysis of this issue, see Yu (2009) and Gros (2017).

25 Srubar provides a classical reading of Husserlian transcendental phenomenology as a metaphysical, idealist, solipsist, and cognitivist approach that collides with current interpretations such as that of Zahavi (2003). Based on Husserl’s later writings, the Danish scholar objects to the idea that transcendental phenomenology constitutes a “classical Cartesian-Kantian subject-philosophy” (2003: 140), emphasizing that, for Husserl, intersubjectivity, corporeality, historicity, and culture play a key role in the transcendental constitution of the world (2003: 133).

26 Andreas Göttlich (2008: 103) poses similar questions.

27 As suggested above, in Luckmann (1979: 199; 1991: 159, 162) the idea of parallelism goes hand in hand with that of a phenomenological “foundation” of sociological research. For him, saying that phenomenology and sociology run parallel does not mean that they have an equal status. The former has a primacy over the latter insofar as it provides its philosophical foundation.

28 Belvedere (2013: 10) also criticizes Luckmann’s use of the metaphor of parallelism but for different reasons.

29 In a similar vein to Merleau-Ponty, Göttlich (2008: 103) and Eberle (2021b; forthcoming) also promote a dialogue of mutual information between phenomenology and empirical social research.

30 For an exhaustive treatment of Luckmann’s argument in “On the Boundaries of the social world,” see Dreher (2012).

31 This reading of “On the Boundaries of the Social World” seems to be compatible with Knoblauch’s (2011: 140f.) claim that there is a “triangulation” between biological sciences, socio-cultural sciences, and phenomenology at work in Luckmann’s thought (see Eberle 2014: 191).
